# Supplementary material for: Glucose‐Sensing Photonic Nanochain Probes with Color Change in Seconds
Source: Adv Sci (Weinh). 2022 Jan 31;9(9):2105239. doi: 10.1002/advs.202105239 (PMC8948609; doi:10.1002/advs.202105239)
Supplement: Supplementary file 1 — Supporting Information [file ADVS-9-2105239-s001.pdf]

ADVANCED SCIENCE
Open Access

Supporting Information

for *Adv. Sci.*, DOI 10.1002/advs.202105239

Glucose-Sensing Photonic Nanochain Probes with Color Change in Seconds

Jinyang Cai, Wei Luo, Juanjuan Pan, Gang Li, Yuyang Pu, Luying Si, Gongpu Shi, Yuxuan Shao, Huiru Ma* and Jianguo Guan**

ADVANCED SCIENCE
Open Access

Supporting Information

for *Adv. Sci.*, DOI: 10.1002/advs202105239

Glucose-sensing photonic nanochain probes with color change in seconds

Jinyang Cai, Wei Luo, Juanjuan Pan, Gang Li, Yuyang Pu, Luying Si, Gongpu Shi, Yuxuan Shao, Huiru Ma*and Jianguo Guan**

Glucose-sensing photonic nanochain probes with color change in seconds

Jinyang Cai, Wei Luo, Juanjuan Pan, Gang Li, Yuyang Pu, Luying Si, Gongpu Shi, Yuxuan Shao, Huiru Ma*and Jianguo Guan**

J. Cai, J, Pan, G. Li, Y. Pu, L. Si, G. Shi, Y. Shao, Prof. J. Guan
State Key Laboratory of Advanced Technology for Materials Synthesis and Processing
International School of Materials Science and Engineering
Wuhan University of Technology
122 Luoshi road, Wuhan 430070, P. R. China
E-mail: guanjg@whut.edu.cn

Dr. W. Luo
School of Materials Science and Engineering
Wuhan University of Technology
122 Luoshi road, Wuhan 430070, P. R. China
E-mail: rowell@whut.edu.cn

Prof. H. Ma
School of Chemistry, Chemical Engineering and Life Science
Wuhan University of Technology
122 Luoshi road, Wuhan 430070, P. R. China
E-mail: mahr@whut.edu.cn

Keywords: photonic crystal nanochains, glucose monitor, response time, colorimetric sensing, preparation mechanism

Abstract: Glucose-sensing photonic crystals are promising for the significant advance of continuous glucose monitoring systems due to the naked-eye colorimetric readouts and non-invasive detection of diabetes, but the long response time hampers their practical applications. Herein, we have for the first time demonstrated probes of photonic nanochains (PNCs) that are capable of continuously and reversibly sensing glucose concentration ([glucose]) variation within seconds by color change without power consumption, much faster by 2~3 orders of magnitude than previous ones. They are comprised of one-dimensional equidistant arrays of magnetic nanoparticles enveloped by tens-of-nanometer-thick phenylboronic acid-functionalized hydrogels, and fabricated by developing selective concentration polymerization of monomers in binary microheterogeneous solvents of dimethyl sulfoxide (DMSO) and H_2O. In this process, both 3-acrylamido phenylboronic acid (AAPBA) and N-2-hydroxyethyl acrylamide (HEAAm) are preferentially dissolved in the small volume of free DMSO concentrated in the vicinity of Fe_3O_4@PVP colloidal nanoparticles, yielding Fe_3O_4@PVP@poly(AAPBA-co-HEAAm) PNCs after UV irradiation under magnetic field. The PNCs in PBS buffer have a wavelength-shift range up to 130 nm when [glucose] changes from 0 to 20 mM. The results can facilitate real-time glucose monitoring, and provide an alternative to produce functional organic-inorganic nanostructures.

Glucose concentration in body fluids is the most important biochemical indicator to diagnose diabetes,[1] loss of consciousness, vascular disease or neurocognitive changes.[2] A variety of glucose probes and sensing technologies have been developed including electrochemical sensors,[3] fluorescent dyes,[1b] and surface plasmon resonance nanoparticles or nanoantenna.[4] Some of them are in practical uses and commercialized. Compared to them, glucose-sensing photonic crystals (PCs) could vary the colors in a wide range of visible spectra following Bragg diffraction due to the volumetric changes with glucose concentration ([glucose]).[5] This colorimetric assay ensures the simplicity and practicality to be read by naked eye as well as the potential application for non-invasive glucose monitoring via urine or tear fluid.[6] Moreover, it is label-free, without external power consumption, not susceptible to electromagnetic fields, and suitable for sterile remote sensing and making wearable devices. These remarkable advantages are expected to enable it to advance continuous glucose monitoring (CGM) systems, which are currently based on subcutaneously inserted electrochemical sensors. These devices generally suffered from signal drift due to electrochemical reaction instability, and thus needed calibration using frequent fingerstick

blood tests, causing obvious pains and inconvenience to the patients on multiple-dose insulin injection or insulin pump therapy.

Up to now, several kinds of glucose-sensing PCs have been developed. For example, with time-consuming dialysis and post-treatment for attaching phenylboronic acid (PBA) moieties, crystalline colloidal arrays (CCAs) of highly charged monodispersed polystyrene nanospheres were implanted in the PBA-modified hydrogel matrix for glucose monitoring.[7] After a tedious stringent process containing template fabrication, monomer infiltration, and the etching process with hazardous hydrofluoric acid or organic solvent, inverse opal PCs are also available for sensing glucose with the assistance of mechanical robust polymers to maintain their structure stability.[8] Hologram-based sensors for detecting glucose have also been fabricated by laser recording techniques, which are costly, sophisticated and hard for mass production.[9] Nevertheless, the so far developed sensing motifs all suffered from long response time, and none of them show real-time continuous glucose monitoring ability, which, however, is necessary for CGM systems to make optimal therapeutic decisions, such as timely insulin delivery.[10] For instance, CCA and holographic sensors normally consume tens of minutes or even hours to reach the equilibrium response due to the relative long diffusion distance of glucose molecules in thick hydrogel (usually at least tens of microns).[7b, 9b] Inverse opal PCs have interconnected voids to facilitate the diffusion of analytes, but still demonstrate slow kinetics and hysteresis as the voids are occupied when the gel is swollen.[11]

Herein, we have for the first time reported probes of glucose-sensing photonic nanochains (PNCs) with response time in seconds by developing selective concentration polymerization of monomers in dimethyl sulfoxide (DMSO)-water microheterogeneous binary solvents. The as-reported PNCs are individual nanochains of 1D periodically arrayed magnetic nanoparticles fixed by tens-of-nm-thick hydrogel shells of poly(3-acrylamido phenylboronic acid–co-N-2-hydroxyethyl acrylamide) (poly(AAPBA-co-HEAAm)). They are able to continuously and reversibly change the structural color with [glucose] within seconds, much faster by 2~3 orders of magnitude than other counterparts due to the significantly reduced diffusion length toward analyte glucose. With less than 40 mol% DMSO in solvents, AAPBA and HEAAm prefer to locate in the vicinity of Fe_3O_4@PVP colloidal nanoparticles, yielding glucose-sensing PNCs after polymerization under magnetic field. The as-obtained PNCs exhibit a structural color frequency-shift range up to 130 nm when [glucose] changes between 0 and 20 mM, achieving the visual detection of [glucose] in a continuous mode. The PNCs could achieve real-time continuous glucose monitoring and the miniaturization of

visual glucose sensors, while the developed polymerization technology may provide an effective route to fabricate functional polymer-based hybrid nanostructures.

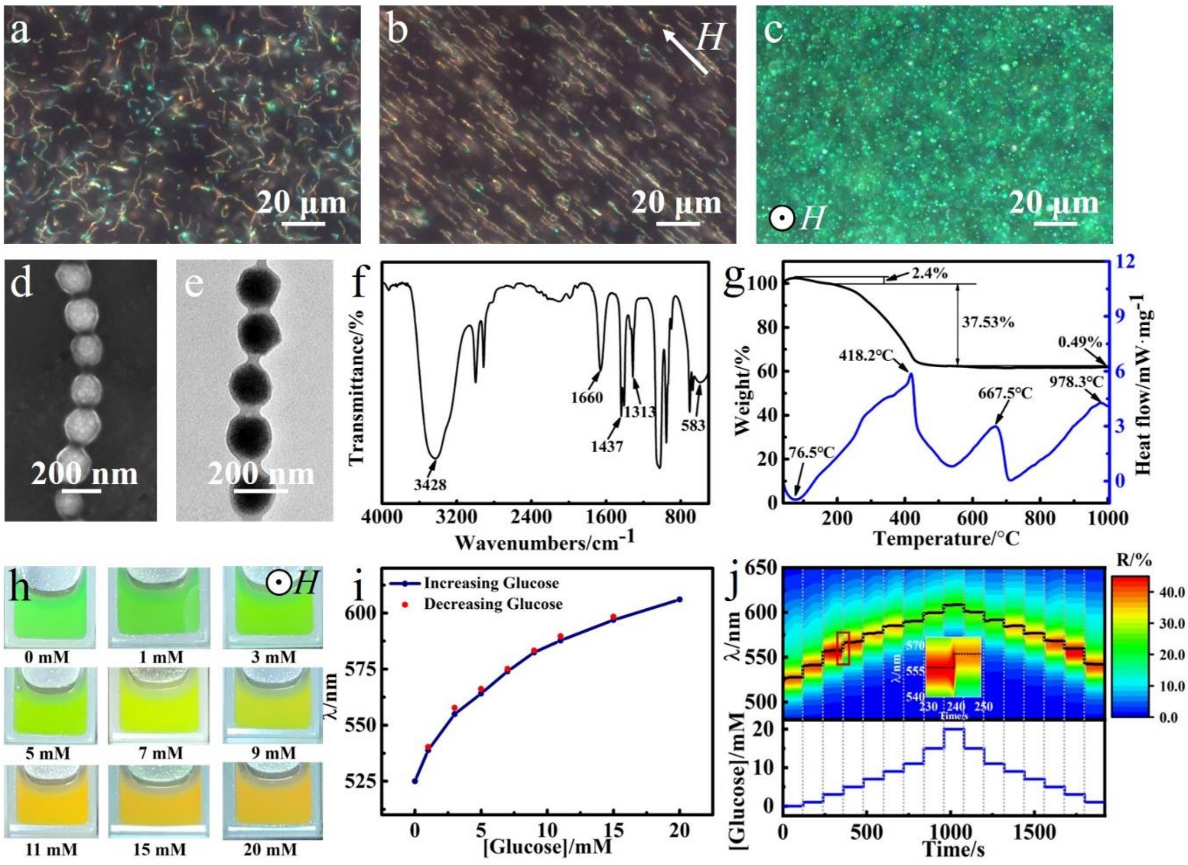

Figure 1. Characterization and glucose-sensing performances of typical Fe_3O_4@PVP@poly(AAPBA-co-HEAAm) PNCs. Dark-field optical microscopy image under (a) zero, (b) a horizontal and (c) a vertical magnetic field. (d) SEM and (e) TEM images, (f) FT-IR spectrum, (g)TG-DSC curves of the typical PNCs. (h) Digital photographs, (i) Diffracted wavelength during a cyclic sweep and (j) Response time of varying [glucose]

Figure 1 illustrates the characterization of the typical Fe_3O_4@PVP@poly(AAPBA-co-HEAAm) PNCs. Almost all of the nanochains presented curved states without magnetic field (Figure 1a), while they gradually became straight parallel to the magnetic field direction with an average length of 10-15 μm (Figure 1b). This indicates their magnetism and flexibility. Figure 1c shows that the nanochains display bright green dots in the dark-field mode when they are aligned parallel to the magnetic field direction. This suggests the structural colors of photonic crystals (PCs). The scanning electron microscopy (SEM) and transmission electron microcopy (TEM) images in Figure 1d-e reveal that the nanochain is a one-dimensional (1D)

ordered particle chain with the same interparticle distance. As reported in previous literature, [12] 1D PNCs diffract light of a specific wavelength as determined by Bragg's law confirming that the bright green colors in Figure 1c stem from the periodical structure of 1D PNCs. In the nanochain, all the magnetic nanoparticles are covered and connected by an organic layer of only about 10~20 nm. The EDS in **Figure S1** also suggests that the inner cores of the nanoparticles are mainly composed of Fe and O elements, and the shell layers mainly contain C, N, O, B elements, which constitute PVP, HEAAm and AAPBA. FT-IR spectrum in **Figure 1f** shows that the absorption peaks at 3428 cm^{-1} and 1660 cm^{-1} are attributed to the N-H group of the acrylamide group (–CONH–) and the stretching vibration of the C=O group of HEAAm, respectively.[13] The peaks at 1437 cm^{-1} and 1313 cm^{-1} are assigned to the stretching vibration peaks of the benzene ring skeleton and the B-O bond of AAPBA.[14] The absorption peak at 583 cm^{-1} is ascribed to Fe-O in Fe_3O_4.[15] From **Figure 1g**, the percentage of Fe_3O_4 and organic substances (PVP and poly(AAPBA-co-HEAAm)) can be calculated to be 59.6 w% and 40.4 w%, respectively. As the percentage of PVP in the Fe_3O_4@PVP magnetic nanoparticles has already known to be 8.8 w% from previous research,[15] the rest poly(AAPBA-co-HEAAm) can be obtained to be 31.6 w% in the PNCs. Furthermore, as the content of B and Fe was measured by the ICP analysis to be 0.30 w% and 28.65 w%, respectively, the weight ratio between Fe and B was 95. Thus, the molar ratio of HEAAm to PAAPBA in the shell layer poly(AAPBA-co-HEAAm) of the PNCs could also be figured out to be 4.8. The above data prove that the prepared 1D PNCs are comprised of poly(AAPBA-co-HEAAm) hydrogel as the shell layer and the Fe_3O_4@PVP nanoparticles as the cores.

Figure 1h depicts that the prepared PNCs have good dispersibility in the PBS buffer solution. With the increment of [glucose] from 0 to 20 mM, their dispersed solution shows the color from green to red. Notably, the color demonstrates a conspicuous transition at around 7 mM, corresponding to the diagnosis indicator of diabetes. [14] Namely, when the solution containing PNCs displays red, [glucose] is above this point, representing the possibility of illness. In contrast, when it turns yellow or green, [glucose] is in the normal fasting blood glucose concentration.[16] **Figure 1i** shows the shifting of the diffraction wavelength (λ) of the PNCs during a cyclic sweep of [glucose] between 0 and 20 mM. The λ shifts red with increasing [glucose]. The shifting range of λ surpasses 80 nm when [glucose] increases from 0 to 20 mM. The λ at each [glucose] both in the increasing and decreasing sweep basically overlapped, proving the good reversibility of the PNCs. **Figure 1j** shows that the optical fiber spectrometer almost synchronously records the equilibrated wavelength of the PNCs when [glucose] changes, suggesting that the response time is within a few seconds. This response

time is at least two to three orders of magnitude shorter than that of the other glucose-sensing PCs, which often take minutes or even hours to reach the equilibrium.[6a, 17] For example, a 10 μm thick polyacrylamide hydrogel film functionalized with AAPBA needed 1.5 h to reach ~90% equilibrium.[17a] The apparent diffusivity of glucose in the hydrogel follows 1-D diffusion equation $\tau = l^2/2D$, where τ, l, D are the time for glucose molecules to reach a steady state, film thickness and the diffusion constant of glucose molecules, respectively. As D in hydrogels is much lower than that in water (5.2×10^{-6} cm^2/s) and was calculated to be about 9.2×10^{-11} cm^2/s,[16] τ for the Fe_3O_4@PVP@poly(AAPBA-co-HEAAm) PNCs is theoretically estimated to be around 20 milliseconds owing to the ultrathin thickness of the hydrogel shell (ca. 20 nm), consistent with our observation. Similar swift responsiveness has also been observed in the responsive hydrogel-based PNCs recently constructed exclusively by hydrophilic monomers.[12b, 12c]

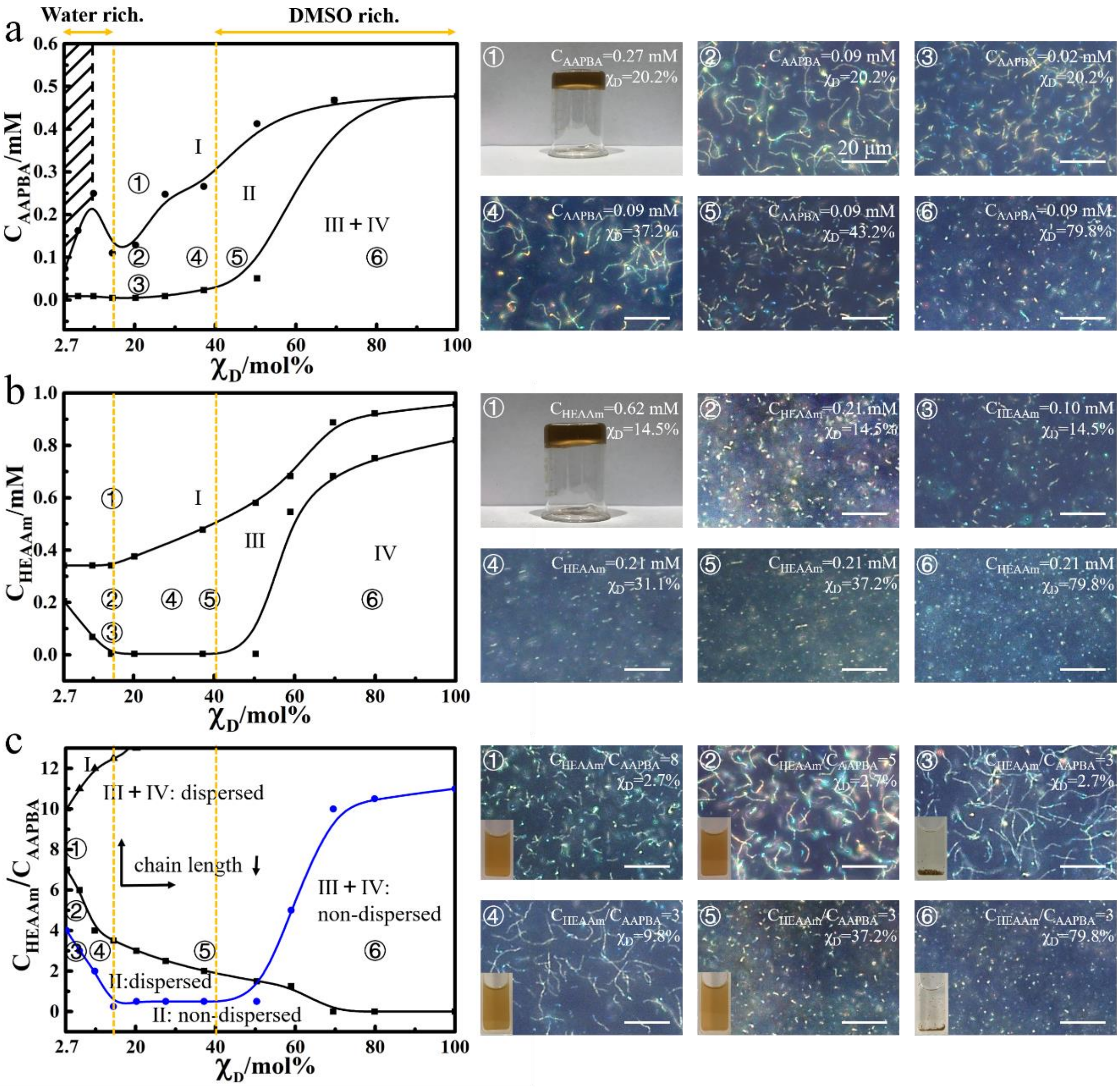

I Hydrogels **II** Nanochains:>5 μm **III** Short-nanochains:<5 μm **IV** Nanoparticles

Figure 2. The phase diagrams and morphologies of the products obtained by the polymerization with Fe_3O_4@PVP nanoparticles in DMSO-H_2O mixed solvents under magnetic field. The phase diagrams (left) and dark-field optical microscope images (right) of the products obtained by the homo-polymerization of (a) AAPBA or (b) HEAAm, and (c) by the copolymerization of AAPBA and HEAAm. χ_D, c_{AAPBA} and c_{HEAAm} represent the molar ratio of DMSO in the DMSO-H_2O mixed solvent, the concentration of AAPBA and HEAAm, respectively. The shadow area in (a) represents where AAPBA cannot dissolve in those mixed solvents. The digital photos on the lower left corner of the microscope images in (c) show the dispersity of the corresponding products in the PBS buffers at room temperature. c_{AAPBA}=0.025 mM in (c).

Previous PNCs are all based on hydrophilic monomers and fabricated by hydrogen bond-guided template polymerization method, where the monomers (or in the assistance of carboxylic acidic polymers) form strong hydrogen bonds with the building blocks of PCs, such as, uniform superparamagnetic Fe_3O_4@PVP nanoparticles.[12] In contrast, the probes of glucose-sensing photonic crystals reported herein are based on relatively hydrophobic 3-acrylamido phenylboronic acid, impossible to obtain following the hydrogen bond-guided template polymerization. In order to decipher the formation mechanism of the glucose-sensing PNCs, the influences of polymerization parameters such as solvent composition, monomer concentration and their ratio etc. on the morphologies and dispersity of the products were respectively studied in **Figure 2**. For the homopolymerization of AAPBA with Fe_3O_4@PVP nanoparticles in DMSO-H_2O mixed solvents under magnetic field, there appear 3 zones with changing the AAPBA concentration (c_{AAPBA}) and χ_D, as shown in Figure 2a. In zone I, the whole prepolymer solution will be transformed into a bulk solid hydrogel without any fluidity after UV irradiation due to the high c_{AAPBA}. With decreasing c_{AAPBA} below a critical value at different χ_D (upper black line in Figure 2a), zone II emerges where the obtained products are nanochains. In this zone, the prepolymer solution after UV polymerization will generate a liquid containing randomly distributed nanochains. The length of these nanochains becomes short with the reduction of c_{AAPBA} or the growth of χ_D until zone (III+IV) appears, in which only short nanochains of 2~3 μm in length or nanoparticles are produced. Interestingly, the length of nanochains drastically decreases when χ_D is more than 40%. This unique phenomenon might be correlated with the microheterogeneity of DMSO aqueous solution. For a DMSO aqueous solution, most DMSO molecules form stable DMSO-water clusters with water molecules via strong hydrogen bonding at a lower χ_D, and fraction

of free DMSO molecules increase abruptly when χ_D is above the eutectic point, corresponding to χ_D of approximately 30~40 mol%.[18] AAPBA is relatively hydrophobic when compared to the other components, and it together with Fe_3O_4@PVP nanoparticles prefers to be dissolved in free DMSO rather than in DMSO-water clusters or free water. Consequently, when χ_D is lower than the eutectic point, the small volume of free DMSO bearing with AAPBA prefer to stay in the vicinity of Fe_3O_4@PVP nanoparticles, while most of free water and DMSO-water clusters are located away from the Fe_3O_4@PVP nanoparticles. In this scenario, monomer molecules AAPBA concentrated in the vicinity of Fe_3O_4@PVP nanoparticles are polymerized under magnetic field and UV irradiation into Fe_3O_4@PVP@PAAPBA nanochains. As evidence, when χ_D=30% and c_{AAPBA} is 10 times lower than its corresponding gel point, Fe_3O_4@PVP@PAAPBA nanochains with an average length around 5~10 μm were still fabricated. This postulation is also supported by the fact that when χ_D is above the eutectic point, there are more volume of free DMSO, in which AAPBA are allowed to be distribute uniformly. This causes to lower the c_{AAPBA} around the Fe_3O_4@PVP nanoparticles. As a result, nanochains diminish.

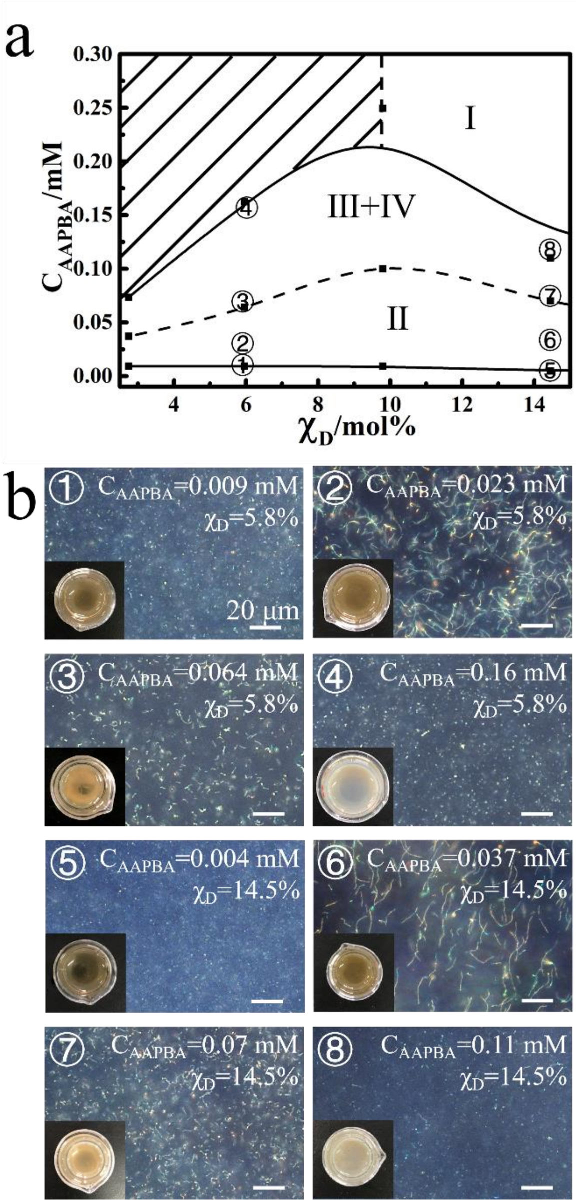

Figure 3. (a) The phase transition of the products obtained by changing c_{AAPBA} when χ_D is in the range of 2.7% and 15%; (b) Dark-field microscope images and optical images (lower left inset) of the products corresponding to those labelled in (a). The shadow area in (a) represents where AAPBA cannot dissolve in those mixed solvents.

Another noteworthy phenomenon is that when χ_D<15%, with the increment of c_{AAPBA}, the obtained nanochains firstly become long, and then get short following a maximum, as depicted in **Figure 3**. Upon increasing c_{AAPBA} in zone Ⅱ, the possibility to form long nanochains is greatly enhanced, in consistence with the growing nanochains when χ_D＞15%. However, further increasing c_{AAPBA} will significantly shorten the chain length. The solubility of excessive PAAPBA in the mixed solvent drops rapidly when χ_D<15%, and they will quickly precipitate out of the mixed solvent making a whitish solution shown in the digital

picture in the lower left corner of Figure S2. Therefore, when the PAAPBA layer on nanochains has not become sufficiently thick and robust to maintain the integrity of nanochains, they will precipitate out together in a form of short nanochains or nanoparticles due to their incompatibility with the solvent.

Since the Fe_3O_4@PVP@PAAPBA nanochains are relatively hydrophobic, they cannot be dispersed in aqueous solution. This impedes their further application for glucose detection. Accordingly, hydrophilic comonomer HEAAm is incorporated to increase the hydrophilicity of the nanochains, prompting their utilization near physiological environment. The influences of HEAAm concentration (c_{HEAAm}) and χ_D on the fabrication of Fe_3O_4@PVP@PHEAAm nanochains are shown in **Figure 2b**. The gelation line for HEAAm is relatively flat compared with that of AAPBA depicted in Figure 2a. Meanwhile, there are only short nanochains with an average length around 2~3 μm appearing in the area encircled by two black lines (zone III) and there is even no appearance of zone II. In contrast, the nanochains obtained by AAPBA are much longer in zone II. Below the black line displays a U-shaped feature, which also rises if χ_D surpasses the eutectic point. However, when χ_D is lower than 15%, higher c_{HEAAm} is also needed to form short nanochains, which make the black line below climb up again. All of the above characteristics might be associated with the hydrophilicity of HEAAm. Specifically, as comonomers HEAAm are miscible with both DMSO and water, they are inclined to be uniformly distributed in the whole prepolymer solution when $\chi_D < 15\%$ (corresponding to water rich region) or $\chi_D > 40\%$ (corresponding to DMSO rich region). Then, higher c_{HEAAm} is needed to form nanochains in these two areas, in accordance with the uprising black line in the lower part of Figure 2b. If $15\% < \chi_D < 40\%$, most DMSO and water form DMSO-H_2O clusters. Thus, relatively low c_{HEAAm} is required to form nanochains in this region. However, the Fe_3O_4@PVP@PHEAAm nanochains are obviously shorter than those acquired by AAPBA. Because even if $15\% < \chi_D < 40\%$, there still exists free water diluting c_{HEAAm} around the Fe_3O_4@PVP nanoparticles surrounded by DMSO molecules.

Figure 2c demonstrates the phase diagram and dispersibility of the products obtained by the copolymerization of AAPBA and HEAAm with Fe_3O_4@PVP nanoparticles in DMSO-H_2O mixed solvents under magnetic field. The regions in the phase diagram are also divided into those belonging to gels, nanochains, short-nanochains, and nanoparticles. The whole gelation line is shown in **Figure S2**. It is very similar to that of the pure HEAAm shown in Figure 2b. This was because only c_{HEAAm} was altered, whereas c_{AAPBA} was fixed at a relatively low level (0.025 mM). Thus, it is c_{HEAAm} that determines the formation of gel in Figure 2c. As the ratio of HEAAm to AAPBA (c_{HEAAm}/c_{AAPBA}) increases, the nanochains gradually become

short, as shown in images ①, ② and ③ of Figure 3c. The hydrophilic transition of the copolymer poly(AAPBA-co-HEAAm) may account for the above results given that the increasing hydrophilicity makes the copolymer more like PHEAAm rather than PAAPBA. As seen in Figure 2a and b, the nanochains obtained by relatively hydrophobic PAAPBA are much longer than those based on hydrophilic HEAAm, provided that PAAPBA does not precipitate out of the solution. This is because monomer AAPBA is mainly dissolved in DMSO preferentially surrounding the Fe_3O_4@PVP nanoparticles, whereas the solvation of AAPBA by water is relatively suppressed. The blue line in Figure 2c is associated with the dispersibility of the prepared products in the PBS 8.0 buffer. Increasing c_{HEAAm} facilitates the dispersion of the obtained nanochains in the buffer solution. Accordingly, only the PNCs fabricated in zone Ⅱ (dispersed) in the phase diagram may possess excellent glucose responsiveness.

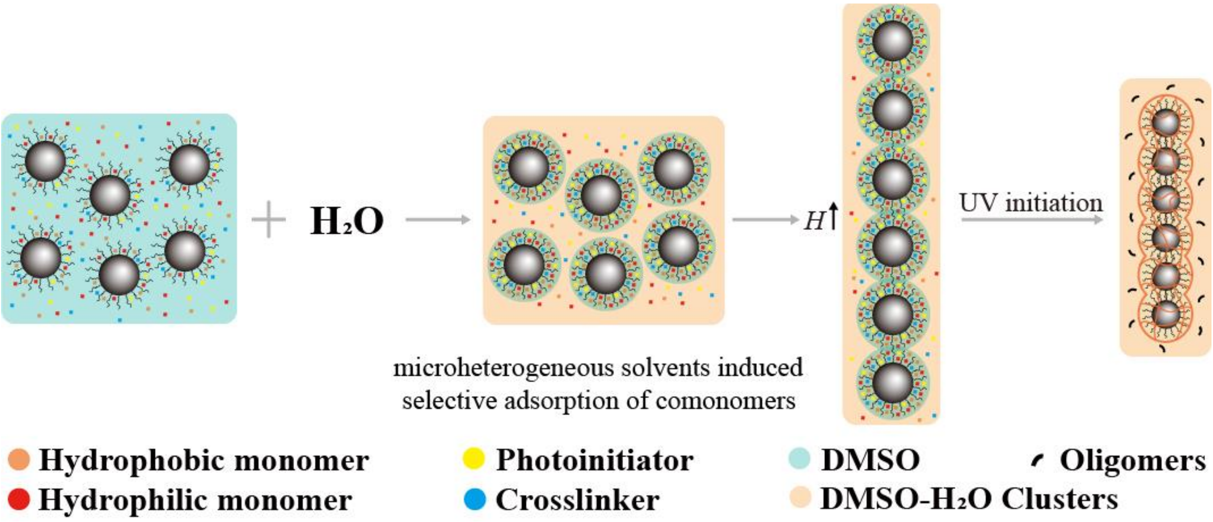

Scheme 1 Formation mechanism of the glucose-sensing PNCs. In the mixed solvent of DMSO and H_2O, most DMSO molecules form DMSO-H_2O clusters via strong hydrogen-bonding interactions besides a small volume of free DMSO. The small volume of free DMSO prefers to stay around Fe_3O_4@PVP nanoparticles and have much better solubility to AAPBA and comonomers than the DMSO-H_2O clusters and free H_2O. Consequently, Fe_3O_4@PVP@poly(AAPBA-co-HEAAm) PNCs are obtained by in-situ polymerization initiated with ultraviolet radiation under a magnetic field.

Based on the above phase diagrams of the products obtained by the polymerization of responsive monomer AAPBA and comonomer HEAAm with monodispersed Fe_3O_4@PVP nanoparticles in DMSO-H_2O mixed solvents under magnetic field, we have proposed the preparation principle of the glucose-sensing PNCs in **Scheme 1**, which is called selective

concentration polymerization of monomers in microheterogeneous solvents. First, the monomers, crosslinking agent, initiator and uniform colloidal Fe_3O_4@PVP nanoparticles are evenly dispersed in DMSO solvent. After adding water, there is strong hydrogen-bonding interactions between DMSO and water to form DMSO-H_2O clusters, meanwhile there was a small amount of free DMSO as well as free water in the solution. According to the analysis in Figure 2, the small volume of free DMSO molecules with relatively hydrophobic AAPBA (the n-octanol/water partition coefficient *log Pow* = 1.787, SDS from Sigma-Aldrich) are more likely to stay around Fe_3O_4@PVP nanoparticles. HEAAm (the n-octanol/water partition coefficient *log Pow* = -0.73, SDS from Sigma-Aldrich) is added as a comonomer to improve the hydrophilicity of the final products. Consequently, the Fe_3O_4@PVP nanoparticles are fixed by poly(AAPBA-co-HEAAm) to form PNCs after the in-situ polymerization has been carried out by ultraviolet radiation under magnetic field.

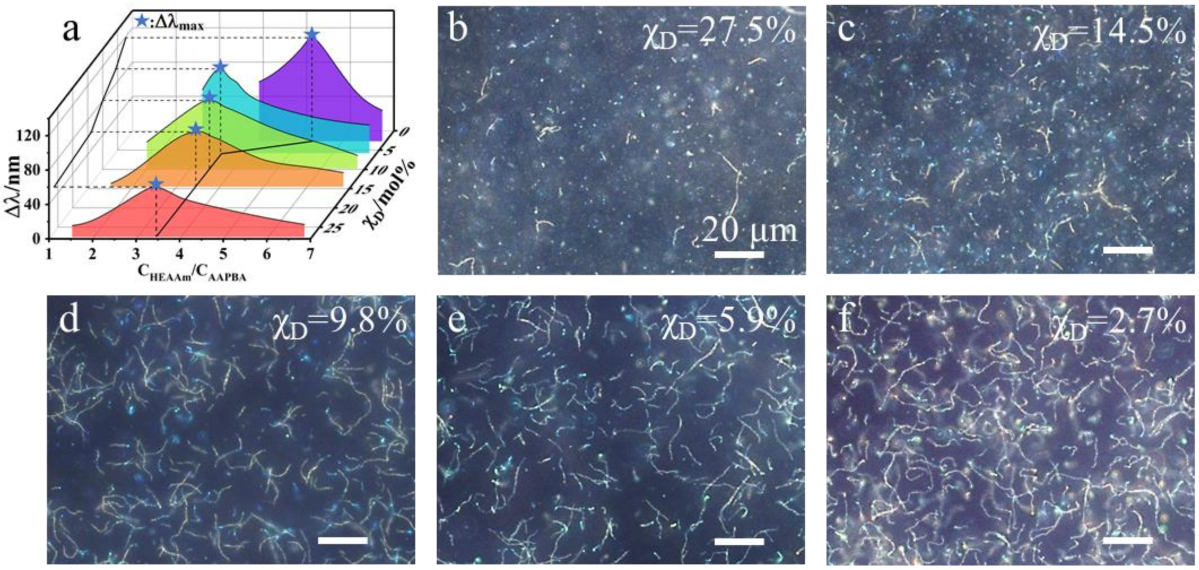

Figure 4. Glucose-sensing capability and morphologies of the Fe_3O_4@PVP@poly (AAPBA-co-HEAAm) PNCs. (a) The diffraction wavelength shift range (Δλ) when [glucose] changes between 0 to 20 mM versus c_{HEAAm}/c_{AAPBA} for the PNCs obtained at different χ_D. (b-f) Dark-field microscope images of the products corresponding to the $\Delta\lambda_{max}$ at each χ_D in (a). c_{AAPBA} = 0.025 mM.

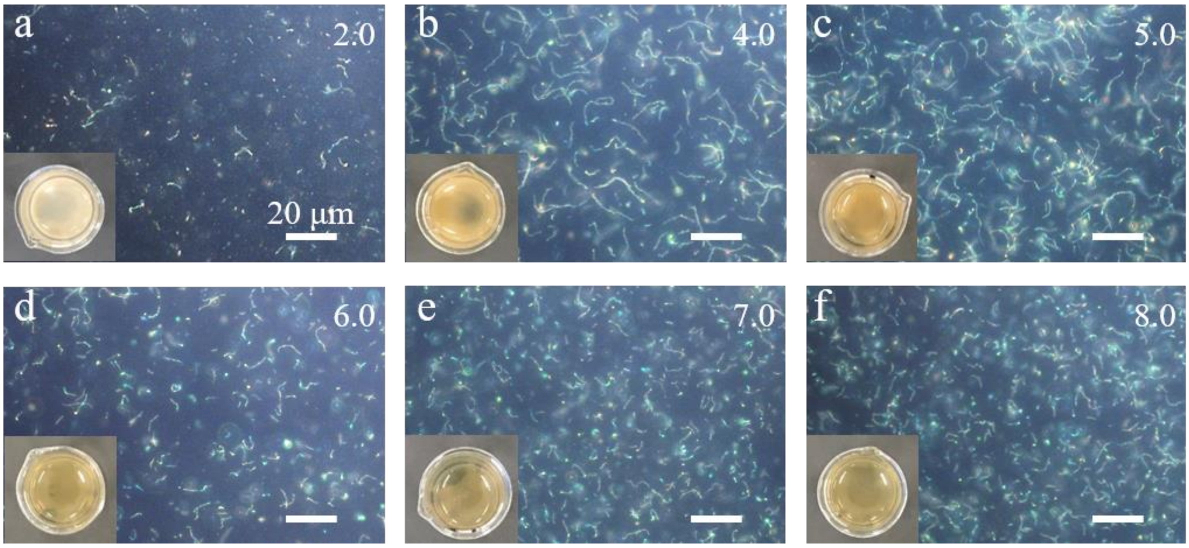

Figure 5. The dark-field microscope images of the nanochains prepared at χ_D = 2.7% and different c_{HEAAm}/c_{AAPBA}. The lower left insets show the optical images of the reacted solution taken after UV polymerization.

In order to elucidate the relationship between the glucose-sensing capabilities of the PNCs and the microstructures, and consequently get the maximum glucose response range of the PNCs, we have fabricated a series of PNCs by varying the feeding ratio of AAPBA and HEAAm (c_{HEAAm}/c_{AAPBA}), as well as χ_D. **Figure 4** shows the diffraction wavelength shift range ($\Delta\lambda$) of the PNCs in PBS buffer when the [glucose] changes between 0 and 20 mM at room temperature under magnetic field. With increasing c_{HEAAm}/c_{AAPBA} at each fixed χ_D, $\Delta\lambda$ of the PNCs firstly rises, and then drops following a maximum value ($\Delta\lambda_{max}$), in accordance with those of the glucose responsive PC films.[19] Typically, the higher the concentration of boronic groups, the greater a response presents. However, excessive AAPBA content will make the hydrogel more hydrophobic, causing it to swell less in PBS buffer solutions. This accounts for the trend of the curves in Figure 3. The comparison of the $\Delta\lambda_{max}$ of samples at each χ_D indicates that when χ_D>5%, all the $\Delta\lambda_{max}$ at each χ_D occur when the c_{HEAAm}/c_{AAPBA} = 3.4. Meanwhile, as χ_D decreases, the $\Delta\lambda_{max}$ at each χ_D gradually increases. The corresponding dark-field microscopic images shown in Figure 3b-f indicate that the chain lengths of those products become longer and longer. This is related to the increase in the thickness of the polymer gel layer around the nanochains, as proved by the change of TG in **Figure S3**. Therefore, it is reasonable to presume that the PNCs contain more glucose responsive hydrogel between neighboring nanoparticles. This hypothesis can also be verified by the initial peak position of those products in PBS buffers without glucose under magnetic field in

Figure S4. Because the interparticle distance is proportional to the peak position according to Bragg's law, and the larger initial peak position of the nanochains in the same dispersion medium represents more poly(AAPBA-co-HEAAm) hydrogel occupying the interparticle distances. Furthermore, the hydrogels acquired at χ_D>5% and c_{HEAAm}/c_{AAPBA}=3.4 almost have an identical composition, indicating an almost equivalent expansion ratio responding to [glucose]. In these circumstances, thicker hydrogel between nanoparticles in the PNCs will induce larger glucose response range. For example, if the interparticle area containing 30 nm thick hydrogel, and the expansion ratio is 1.5, the actual swelling distance is greater than 20 nm thick hydrogel ($\Delta d_{30} = 30 \times 0.5 > \Delta d_{20} = 20 \times 0.5$). When χ_D<5%, the c_{HEAAm}/c_{AAPBA} corresponding to $\Delta\lambda_{max}$ are no longer at 3.4 but shift to 5.3. This is caused by the transition of dispersity of the polymer in the mixed solvent. As it can be seen in Figure 2c, if χ_D is lower than 5%, the products prepared at c_{HEAAm}/c_{AAPBA}=3.4 are out of zone Ⅱ (dispersed) in the phase diagram resulting in the agglomeration of nanochains as well as the aforementioned whitening phenomenon during the polymerization (polymer together with short nanochains or nanoparticles quickly precipitate out of the solution). Consequently, the content of HEAAm is increased to avoid whitening of the solution during polymerization and obtain longer nanochains to achieve large glucose responsiveness (**Figure 5**). Among all the samples, the PNCs obtained at χ_D = 2.7% and c_{HEAAm}/c_{AAPBA} = 5.3 have the longest chain length, and their glucose-responsive range is up to 130 nm.

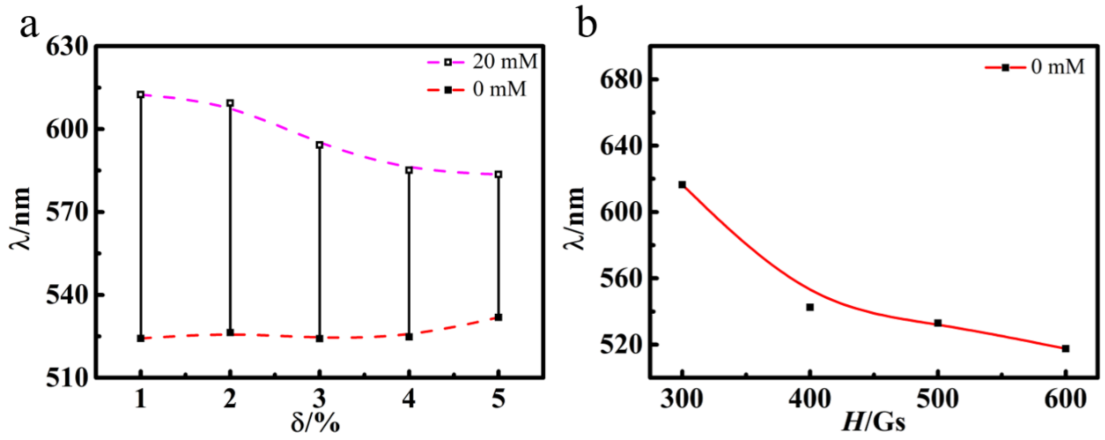

Figure 6. The diffraction wavelength of Fe_3O_4@PVP@poly(AAPBA-co-HEAAm) PNCs versus (a) the amount of added crosslinking agent (δ) and (b) magnetic field strengths (*H*) used in the polymerization.

Figure 6 shows the relationship between the diffraction wavelength of the Fe_3O_4@PVP@poly(AAPBA-co-HEAAm) PNCs at different [glucose] and the preparation

conditions including the amount of the crosslinking agent (δ) and polymerization magnetic field (*H*). The rising δ brings the increase in the crosslinking degree and thus the rigidity of PNCs. Consequently, the swelling or shrinking capability of their hydrogel coatings is limited along with the glucose-response ranges (Figure 4a). This is confirmed by the consecutively descending peak positions of the PNCs at [glucose] of 20 mM with increasing δ. The above tendency is similar to that of our previous pH responsive PNCs, but the two systems show great differences in the extent of the peak position variation induced by adding the same δ. For example, when δ changed from 2% to 4%, the pH responsive PNCs maintained less than 60% of its original response range at 2%. In contrast, the glucose responsive PNCs hold 75% of the response range at the same condition. This is because the pH responsive PNCs adopted more hydrophobic ethylene glycol dimethacrylate (EGDMA) as the crosslinking agent, while the glucose responsive PNCs utilized hydrophilic BIS. According to the mechanism in this paper, hydrophilic BIS may distribute more evenly in DMSO-H_2O mixtures making them less efficient to concentrate around Fe_3O_4@PVP nanoparticles. In fact, if BIS was substituted by EGDMA in the preparation procedure of glucose responsive PNCs, much less crosslinker is needed to produce the same effects of contraction. However, EGDMA also raises the hydrophobicity of the PNCs deteriorating their dispersity in aqueous buffer solutions.

The influence of the *H* used in the polymerization process on the diffraction wavelength of the PNCs has further been examined. As *H* increases, the obtained PNCs show blue shifted λ in the PBS buffer solution without glucose (Figure 4b). It is easy to understand that strong *H* will compress the spacing of nanoparticles during the polymerization causing shorter wavelengths to be selected for reflection. Thus, the visually perceptible response ranges of the PNCs to [glucose] could be readily modulated by adjusting both λ and *H*.

In conclusion, we have demonstrated a new category of optical responsive probes capable of rapidly, continuously and reversibly sensing glucose concentration based on structural color variation, Fe_3O_4@PVP@poly(AAPBA-co-HEAAm) PNCs. The PNCs are wrapped by only tens of nanometer thick hydrogel shell, prompting the fast diffusion of glucose molecules and significantly reduced response time to a few seconds, at least two orders of magnitude better than their previous counterparts. They are obtained by selective concentration polymerization of monomers in DMSO-H_2O microheterogeneous binary solvents. The morphology evolution and the formation mechanism of the products were systematically interrogated depending on the χ_D of the binary solvents as well as the concentration of monomers. When $\chi_D < 40\%$, most DMSO will form hydrogen-bonding

clusters with water, and the populations of free DMSO drop greatly. Relatively hydrophobic AAPBA along with little free DMSO molecules have a tendency to reside in the periphery of the Fe_3O_4@PVP nanoparticles, which amplified the microheterogeneity of the monomer distribution. Thus, PNCs are obtained after polymerization under magnetic field. The incorporation of HEAAm into the hydrogel increases the dispersibility of the PNCs in PBS buffer solution. The maximum diffraction peak-shift range ($\Delta\lambda_{max}$) of the PNCs is closely related to the content of poly(AAPBA-co-HEAAm) hydrogel in the PNCs, which is inversely proportional to χ_D used in polymerization. The PNCs show an optical spectra wavelength–shift range up to 130 nm when [glucose] changed from 0 to 20 mM, benefiting for naked-eye detection. It is expected that the as-developed glucose sensing PNCs enable the real-time continuous glucose monitoring as well as the miniaturization of glucose diagnosis devices. The developed selective concentration polymerization of monomers in microheterogeneous solvents may pave a way to construct functional polymer-inorganic hybrid nanostructures.

Experimental Section

Materials: 3-Acrylamido phenylboronic acid (AAPBA) was purchased from Sigma-Aldrich. N-(2-Hydroxyethyl) acrylamide (HEAAm), N,N'-methylenebisacrylamide (BIS), 2-Hydroxy-2-methylpropiophenone (HMPP) were obtained from Aladdin Co. Ltd. Dimethyl sulfoxide (DMSO), D(+)-glucose were purchased from Sinopharm Chemical Reagent Co. Ltd，China. All of the above reagents were used as received without further purification. Deionized water (Aquapro) was used for the experiment. Superparamagnetic Fe_3O_4@PVP colloidal nanoparticles with an average particle size of 150 nm were synthesized by a one-pot solvothermal polyol process according to our previous report, and stored in ethanol for further use (10 mg/ml).[15]

Preparation of prepolymerization solution: The stock solution of Fe_3O_4@PVP colloidal nanoparticles was obtained by centrifuging their ethanol solution (1 mL) and then redispersing it into DMSO (0.455 mL) under sonication. The stock solutions of monomers or photo-initiators were produced by dissolving AAPBA (0.5 mM), HEAAm (4.25 mM), BIS (0.1 mM) and HMPP (0.2 mM) in 545 μL, 181 μL, 500 μL and 500 μL DMSO, respectively.

Preparation of Fe_3O_4@PVP@PAAPBA photonic nanochains: The Fe_3O_4@PVP colloidal nanoparticles stock solution (12 μL), AAPBA stock solution (27.5 μL), HMPP stock solution (2 μL), DMSO (58.6 μL) and deionized water (900 μL) were firstly mixed in a 10 mL glass beaker to form a prepolymer solution by sonication. Then, the beaker was placed above the

center of a 10×10×2 cm NdFeB square magnet with a distance of 2.0 cm (500 Gs) for 120 s. Subsequently, the UV light was turned on for curing (5 minutes). Afterwards, 3ml DMSO was added into the beaker to dilute the solution. Finally, the products of Fe_3O_4@PVP@PAAPBA PNCs were magnetically separated from the solution and re-dispersed in DMSO for storing. A variety of products were obtained by changing the concentration of AAPBA or χ_D in the prepolymer solution.

Preparation of Fe_3O_4@PVP@PHEAAm photonic nanochains: The Fe_3O_4@PVP colloidal nanoparticles stock solution (12 μL), HEAAm stock solution (18.5 μL), BIS stock solution (13 μL), HMPP stock solution (10 μL), DMSO (60 μL) and deionized water (900 μL) were firstly mixed in a 10 mL glass beaker to form a prepolymer solution by sonication. Then, the beaker was placed above the center of a 10×10×2 cm NdFeB square magnet with a distance of 2.0 cm (500 Gs) for 120 s, and subsequently irradiated with the UV light for 5 minutes. Afterwards, 3ml DMSO was added into the beaker to dilute the solution. Finally, the products of Fe_3O_4@PVP@PHEAAm PNCs were magnetically separated from the solution and re-dispersed in DMSO for storing. Various products were obtained by changing the concentration of HEAAm or χ_D in the prepolymer solution.

Preparation of Fe_3O_4@PVP@poly(AAPBA-co-HEAAm) photonic nanochains: The Fe_3O_4@PVP colloidal nanoparticles stock solution (12 μL), AAPBA stock solution (27.5 μL), HEAAm stock solution (18.5 μL), BIS stock solution (15.5 μL), HMPP stock solution (12 μL), DMSO (28 μL) and deionized water (900 μL) were firstly mixed in a 10 mL glass beaker to form a prepolymer solution by sonication. Then, the beaker was placed above the center of a 10×10×2 cm NdFeB square magnet with a distance of 2.0 cm (500 Gs) for 120 s, and subsequently illuminated by the UV light for 5 minutes. Afterwards, 3ml DMSO was added into the beaker to dilute the solution. Finally, the products of Fe_3O_4@PVP@poly(AAPBA-co-HEAAm) PNCs were magnetically separated from the solution and re-dispersed in DMSO for storing. Various products were obtained by changing the concentration of monomers or χ_D in the prepolymer solution.

Characterization: All digital photos were taken by using iPhone mobile phone. A JEM-2100F transmission electron microscope (TEM) instrument (JEOL, Japan) was used to capture TEM images with an acceleration voltage of 200 kV. The field-emission scanning electron microscopy (FE-SEM) images were collected on a Hitachi S-4800 scanning electron microscope. A 60-SXBFTIR spectrometer was used to collect Fourier transform infrared (FTIR) spectra in the range of 400 to 4000 cm^{-1} with a resolution of 4 cm^{-1}. A NETZSCH-STA449C/G instrument was used to conduct thermal analysis in air at room temperature to

1000 °C and heating rate of 10 °C min^{-1}. The reflectance spectra were all obtained by using a fiber optic spectrometer USB 2000+. All the dark-field microscope images were captured by dispersing nanochains in DMSO solution and then recorded by an optical microscope (Zeiss Axio Observer 5M, Germany).

Glucose detection: Firstly, a PBS buffer with pH=8.0 and physiological ionic strength (150 mM) was prepared according to a previous study.[6a] During the test, the PNCs were dispersed in the above PBS buffer, then, adding a small amount of high-concentrated glucose solution (1600 mM) to adjust its concentration. After shaking for 5 s, it was placed on a 200 Gs magnetic field, and the diffraction spectrum was detected along the magnetic field direction by a fiber optic spectrometer. All the tests were performed at room temperature.

Supporting Information

Supporting Information is available from the Wiley Online Library or from the author.

Acknowledgements

This work was financially supported by the National Natural Science Foundation of China (51 521001, 51573144), the Natural Science Foundation of Hubei Province (2019CFB572), the Fundamental Research Funds for the Central Universities (WUT: 2020III 022).

Conflict of Interest

The authors declare no conflict of interest.

Received: ((will be filled in by the editorial staff))
Revised: ((will be filled in by the editorial staff))
Published online: ((will be filled in by the editorial staff))

References

[1] a) P. Z. Zimmet, D. J. Magliano, W. H. Herman, J. E. Shaw, *Lancet Diabetes Endocrinol.* **2014**, *2* (1), 56; b) Q. Wu, L. Wang, H. Yu, J. Wang, Z. Chen, *Chem. Rev.* **2011**, *111* (12), 7855.

[2] a) L. A. DiMeglio, C. Evans-Molina, R. A. Oram, *The Lancet* **2018**, *391* (10138), 2449; b) S. Chatterjee, K. Khunti, M. J. Davies, *The Lancet* **2017**, *389* (10085), 2239,.

[3] D. Rodbard, *Diabetes Technol. Ther.* **2016**, *18* (S2), S2.

[4] a) M. Zhang, G. Qing, C. Xiong, R. Cui, D.-W. Pang, T. Sun, *Adv. Mater.* **2013**, *25* (5), 749; b) H. He, X. Xu, H. Wu, Y. Jin, *Adv. Mater.* **2012**, *24* (13), 1736; c) M. Mesch, C. Zhang, P. V. Braun, H. Giessen, *ACS Photonics* **2015**, *2* (4), 475.

[5] T. Elshaarani, H. Yu, L. Wang, A. Zain ul, R. S. Ullah, M. Haroon, R. U. Khan, S. Fahad, A. Khan, A. Nazir, M. Usman, K.-u.-R. Naveed, *J. Mat. Chem. B* **2018**, *6* (23), 3831.

[6] a) M. Elsherif, M. U. Hassan, A. K. Yetisen, H. Butt, *ACS Nano* **2018**, *12* (6), 5452; b) V. L. Alexeev, S. Das, D. N. Finegold, S. A. Asher, *Clin. Chem.* **2004**, *50* (12), 2353; c) Z. Yan, M. Xue, Q. He, W. Lu, Z. Meng, D. Yan, L. Qiu, L. Zhou, Y. Yu, *Anal. Bioanal. Chem.* **2016**, *408* (29), 8317.

[7] a) C. Zhang, G. G. Cano, P. V. Braun, *Adv. Mater.* **2014**, *26* (32), 5678; b) C. Zhang, M. D. Losego, P. V. Braun, *Chem. Mat.* **2013**, *25* (15), 3239; c) M. M. Ward Muscatello, L. E. Stunja, S. A. Asher, *Anal. Chem.* **2009**, *81* (12), 4978; d) M. Ben-Moshe, V. L. Alexeev, S. A. Asher, *Anal. Chem.* **2006**, *78* (14), 5149; e) V. L. Alexeev, A. C. Sharma, A. V. Goponenko, S. Das, I. K. Lednev, C. S. Wilcox, D. N. Finegold, S. A. Asher, *Anal. Chem.* **2003**, *75* (10), 2316.

[8] a) M. Honda, K. Kataoka, T. Seki, Y. Takeoka, *Langmuir* **2009**, *25* (14), 8349; b) Y.-J. Lee, S. A. Pruzinsky, P. V. Braun, *Langmuir* **2004**, *20* (8), 3096.

[9] a) N. Jiang, H. Butt, Y. Montelongo, F. Liu, S. Afewerki, G.-L. Ying, Q. Dai, S.-H. Yun, A. K. Yetisen, *Adv. Funct. Mater.* **2018**, *28* (24), 1702715; b) A. K. Yetisen, Y.

Montelongo, F. da Cruz Vasconcellos, J. L. Martinez-Hurtado, S. Neupane, H. Butt, M. M. Qasim, J. Blyth, K. Burling, J. B. Carmody, M. Evans, T. D. Wilkinson, L. T. Kubota, M. J. Monteiro, C. R. Lowe, *Nano Lett.* **2014**, *14* (6), 3587; c) S. Kabilan, A. J. Marshall, F. K. Sartain, M.-C. Lee, A. Hussain, X. Yang, J. Blyth, N. Karangu, K. James, J. Zeng, D. Smith, A. Domschke, C. R. Lowe, *Biosens. Bioelectron.* **2005**, *20* (8), 1602.

[10] a) S. P. Nichols, A. Koh, W. L. Storm, J. H. Shin, M. H. Schoenfisch, *Chem. Rev.* **2013**, *113* (4), 2528; b) D. C. Klonoff, *Roadmap for 21st century diabetes therapy* **2005**, *28* (5), 1231.

[11] Y.-J. Lee, C. E. Heitzman, W. R. Frei, H. T. Johnson, P. V. Braun, *J. Phys. Chem. B* **2006**, *110* (39), 19300.

[12] a) Y. Hu, L. He, Y. Yin, *Angew. Chem.-Int. Edit.* **2011**, *50* (16), 3747; b) L. Kong, Y. Feng, W. Luo, F. Mou, K. Ying, Y. Pu, M. You, K. Fang, H. Ma, J. Guan, *Adv. Funct. Mater.* **2020**, *30* (52); c) W. Luo, Q. Cui, K. Fang, K. Chen, H. Ma, J. Guan, *Nano Lett.* **2020**, *20* (2), 803; d) M. Wang, L. He, Y. Hu, Y. Yin, *J. Mater. Chem. C* **2013**, *1* (38), 6151; e) Y. Liu, Q. Fan, G. Zhu, G. Shi, H. Ma, W. Li, T. Wu, J. Chen, Y. Yin, J. Guan, *Mater. Horizons* **2021**, 8, 2032.

[13] X. Hong, Y. Peng, J. Bai, B. Ning, Y. Liu, Z. Zhou, Z. Gao, *Small* **2014**, *10* (7), 1308.

[14]Y. Zhong, B. Song, D. He, Z. Xia, P. Wang, J. Wu, Y. Li, *Nanotechnology* **2020**, *31* (39), 395601.

[15] W. Luo, H. Ma, F. Mou, M. Zhu, J. Yan, J. Guan, *Adv. Mater.* **2014**, *26* (7), 1058.

[16] C. W. Pirnstill, B. H. Malik, V. C. Gresham, G. L. Coté, *Diabetes Technol. Ther.* **2012**, *14* (9), 819.

[17] a) A. K. Yetisen, Y. Montelongo, F. da Cruz Vasconcellos, J. L. Martinez-Hurtado, S. Neupane, H. Butt, M. M. Qasim, J. Blyth, K. Burling, J. B. Carmody, M. Evans, T. D. Wilkinson, L. T. Kubota, M. J. Monteiro, C. R. Lowe, *Nano Lett.* **2014**, *14* (6), 3587; b) C. Zhang, G. G. Cano, P. V. Braun, *Adv. Mater.* **2014**, *26* (32), 5678; c) C. Chen, Z.-Q.

Dong, J.-H. Shen, H.-W. Chen, Y.-H. Zhu, Z.-G. Zhu, *ACS Omega* **2018**, *3* (3), 3211; d) F. Xue, Z. Meng, F. Wang, Q. Wang, M. Xue, Z. Xu, *J. Mater. Chem. A* **2014**, *2* (25), 9559.

[18] a) K. I. Oh, K. Rajesh, J. F. Stanton, C. R. Baiz, *Angew. Chem.-Int. Edit.* **2017**, *56* (38), 11375; b) D. N. Shin, J. W. Wijnen, J. B. F. N. Engberts, A. Wakisaka, *J. Phys. Chem. B* **2001**, *105* (29), 6759.

[19] S. Kabilan, A. J. Marshall, F. K. Sartain, M. C. Lee, A. Hussain, X. Yang, J. Blyth, N. Karangu, K. James, J. Zeng, D. Smith, A. Domschke, C. R. Lowe, *Biosens. Bioelectron.* **2005**, *20* (8), 1602.

Table of contents entry

Selective concentration polymerization of monomers in microheterogeneous solvents was developed for the fabrication of glucose-sensing photonic nanochain probes, which can be used to quantitatively detect glucose concentration without power consumption via color changes within seconds, at least 2~3 orders of magnitude faster than those of the previous counterparts due to the significantly reduced diffusion length.

Keywords: photonic crystal nanochains, glucose monitor, response time, colorimetric sensing, preparation mechanism

J. Cai, W. Luo*, J. Pan, G. Li, Y. Pu, L. Si, G. Shi, Y. Shao, H. Ma*, J. Guan*

Title
Glucose-sensing photonic nanochain probes with color change in seconds

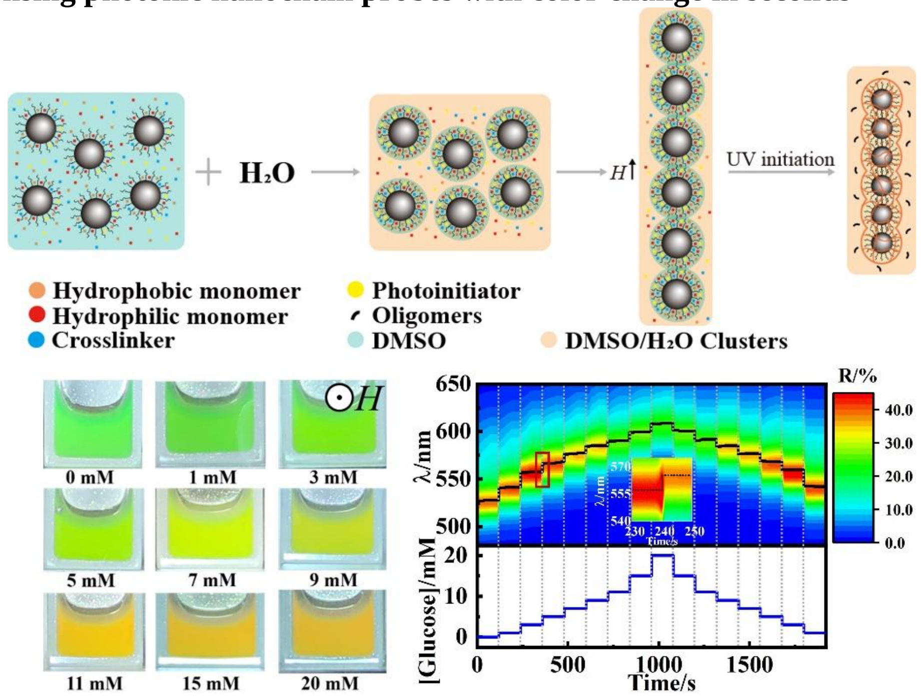

ToC

Supporting Information

Glucose-sensing photonic nanochain probes with color change in seconds

Jinyang Cai, Wei Luo*, Juanjuan Pan, Gang Li, Yuyang Pu, Luying Si, Gongpu Shi, Yuxuan Shao, Huiru Ma*and Jianguo Guan*

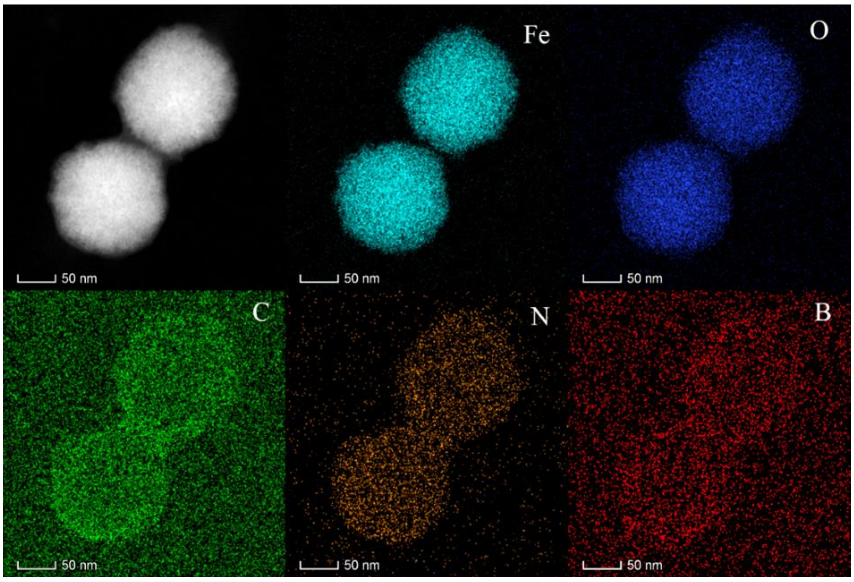

Figure S1. EDS mapping of the as-obtained Fe_3O_4@PVP@poly(AAPBA-co-HEAAm) PNCs.

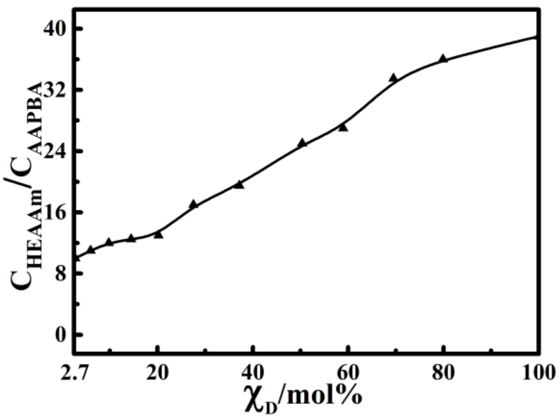

Figure S2. The full version of the gel transition line in Figure 2c by the copolymerization of AAPBA and HEAAm with different χ_D.

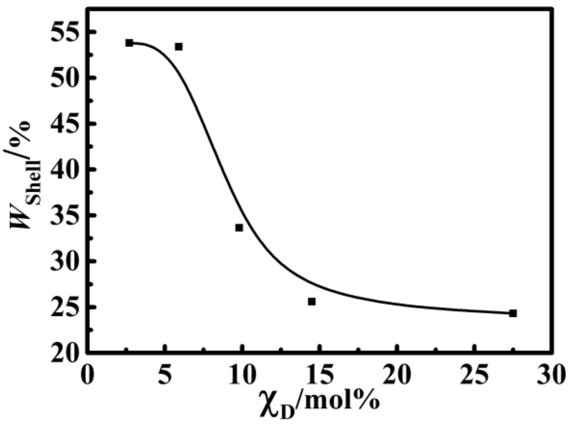

Figure S3. The effect of χ_D on the organic shell percentage (W_{Shell}) in the PNCs corresponding to $\Delta\lambda_{max}$.

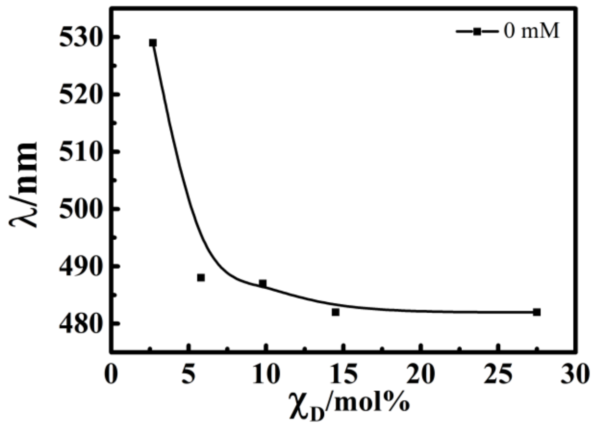

Figure S4. The effect of χ_D on the initiate diffraction wavelength (λ) of the PNCs corresponding to $\Delta\lambda_{max}$.
